# In-Depth Characterization of Apoptosis N-Terminome Reveals a Link Between Caspase-3 Cleavage and Posttranslational N-Terminal Acetylation

**DOI:** 10.1016/j.mcpro.2023.100584

**Published:** 2023-05-24

**Authors:** Rawad Hanna, Andrey Rozenberg, Layla Saied, Daniel Ben-Yosef, Tali Lavy, Oded Kleifeld

**Affiliations:** Faculty of Biology, Technion-Israel Institute of Technology, Haifa, Israel

**Keywords:** LysN, peptidyl-Lys metalloendopeptidase, N-terminomics, N-terminal acetylation, caspase-3, degradomics

## Abstract

The N termini of proteins contain information about their biochemical properties and functions. These N termini can be processed by proteases and can undergo other co- or posttranslational modifications. We have developed LATE (LysN Amino Terminal Enrichment), a method that uses selective chemical derivatization of α-amines to isolate the N-terminal peptides, in order to improve N-terminome identification in conjunction with other enrichment strategies. We applied LATE alongside another N-terminomic method to study caspase-3-mediated proteolysis both *in vitro* and during apoptosis in cells. This has enabled us to identify many unreported caspase-3 cleavages, some of which cannot be identified by other methods. Moreover, we have found direct evidence that neo-N-termini generated by caspase-3 cleavage can be further modified by Nt-acetylation. Some of these neo-Nt-acetylation events occur in the early phase of the apoptotic process and may have a role in translation inhibition. This has provided a comprehensive overview of the caspase-3 degradome and has uncovered previously unrecognized cross talk between posttranslational Nt-acetylation and caspase proteolytic pathways.

## Highlights

• New N-terminomics method called LATE based on LysN and α-amine specific labeling.
• LATE combined with common N-terminomics, significantly improved N-termini coverage.
• Combined methods revealed many unreported caspase-3 cleavages during apoptosis.
• A first report of post translational Nt-acetylation at caspase generated neo-termini

## Graphical Abstract

**Figure:**
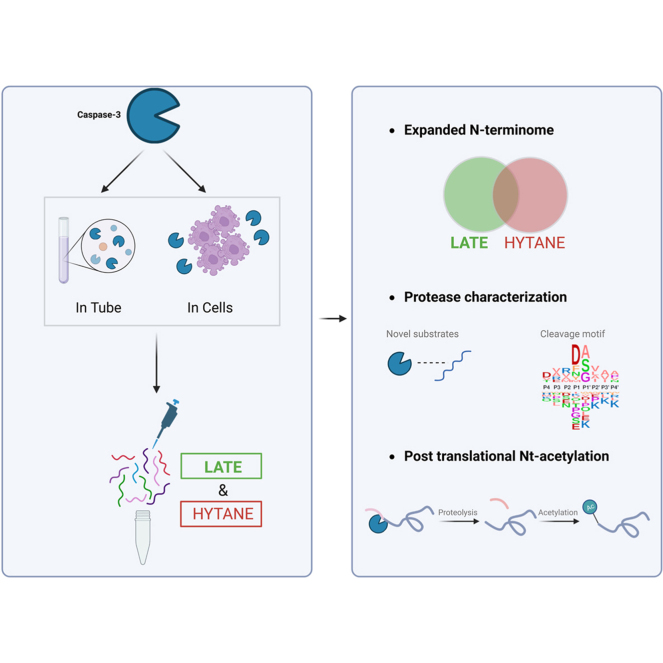

## In Brief

Protein N termini contain information about their biochemical properties. We developed a new method to study N-terminomics called LATE that provides information that cannot be obtained by other methods. When LATE was combined with another method called HYTANE, we discovered new substrates and cleavage sites of caspase-3 during apoptosis. We found that some of the new N termini generated by caspase-3 during apoptosis can undergo N-terminal acetylation.

Proteases used to be described as degradative enzymes that disassemble unwanted or damaged proteins to generate amino acids for the cell (1, 2). Many proteases, however, are very precise and specific enzymes, and their activity produces new polypeptides for different purposes (1, 3). Such specific proteolytic cleavages are irreversible posttranslational modifications. They are crucial for cellular processes and therefore tightly regulated (4, 5, 6, 7). Thus, characterization of protease activity and substrate specificity are essential for completing the functional annotation of any proteome.

Mass spectrometry (MS)-based proteomics is one of the most commonly used methods for system-wide protein studies. Despite this, there are plentiful challenges to overcome when MS is used to identify and quantify proteolytic substrates (8). These challenges predominantly stem from the low abundance of proteolytic fragments, which can easily be masked by other peptides. Moreover, background *in vivo* proteolysis events and the transient state of protein synthesis and degradation also contribute to the system complexity when studying a specific protease activity or treatment-directed proteolysis. Over the past decades, various approaches have been developed for reliable detection of proteolytic fragments by separating them from the background proteome (9). N-terminomics methods are used to isolate and characterize the N-terminal fragment of every protein (N-terminome), usually by utilizing a series of chemical reactions to manipulate the desired subgroup. Negative selection methods such as combined fractional diagonal chromatography (10), terminal amine isotopic labeling of substrates (11), and hydrophobic tagging-assisted N-termini enrichment (HYTANE) (12) are based on the depletion of internal peptides to enhance the identification of proteolytic products N terminus (neo N-termini) and original N termini, including naturally modified ones. Positive selection techniques are based on the enrichment of protein fragments containing unblocked (or free) N termini after tagging them with biotin (13) and therefore cannot be used to study the majority of eukaryotic N-terminal peptides that are naturally blocked (13, 14).

Each method has advantages and limitations weighted by its reliability, sensitivity, availability, cost, and time consumption. For example, combined fractional diagonal chromatography can be applied to study the N-terminome with low sequence biases using commercially available reagents, but it requires a long analysis time and therefore extensive instrumental usage, which can make it difficult to analyze many samples at once (9, 10). Terminal amine isotopic labeling of substrates, on the other hand, is simpler and more straightforward but requires the use of a specific polymer (11, 15). A recently developed method based on hydrophobic tagging akin to HYTANE, termed high-efficiency undecanal-based N-termini enrichment (16), utilizes cheaper reagents and has shown to be useful for low-protein-amount samples. Most of the negative selection N-terminomics approaches require primary amine labeling and thus involve blocking of lysine ε-amines. As a result, these methods cannot use proteases with lysine cleavage specificity, which significantly restricts their application in annotation of the N-terminome.

The N terminus of the nascent polypeptide chain as translated from its open reading frame (ORF) initiation site is the first to undergo modifications. Such modifications include N-terminal methionine excision and acetylation, which usually take place cotranslationally when the protein is still short and bound to the ribosome (17). For the majority of proteins, the N-terminal methionine is removed by methionine aminopeptidases (18). Nt-acetylation is catalyzed by N-terminal acetyltransferases, which utilize acetyl-CoA as the donor for the transfer of the activated acetyl moiety to the N terminus of the protein (19). It is estimated that ∼80% of human proteins are modified by N-terminal acetylation (Nt-acetylation). By altering the N-terminal charge and hydrophobicity of a protein, Nt-acetylation can affect a range of its properties including stability, folding, protein–protein interactions, and subcellular targeting (19).

**Fig. 1:**
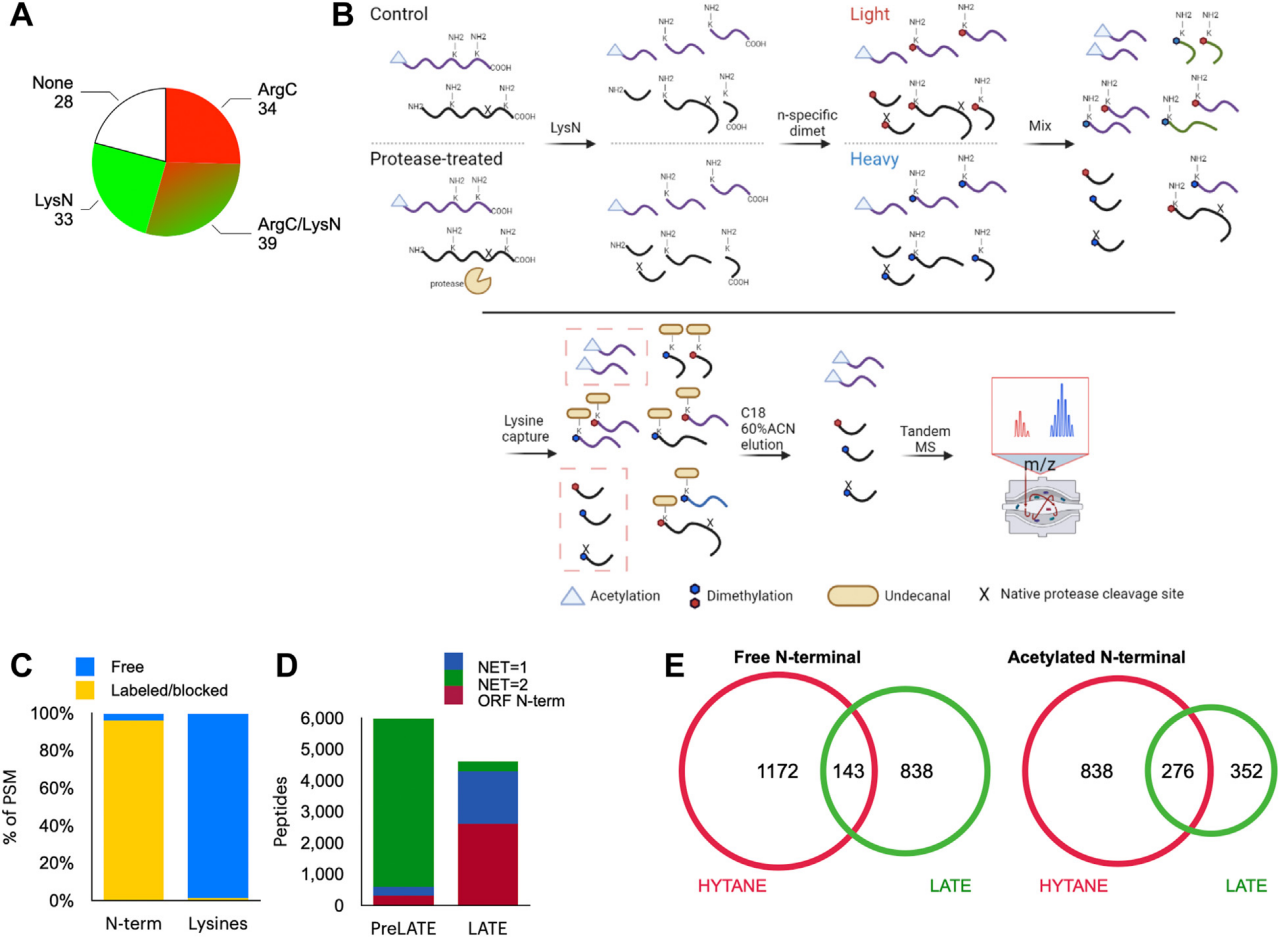
**LysN Amino Terminal Enrichment (LATE) workflow for improved N-terminome coverage.***A*, *in silico* analysis of the “Identifiable” and “Not identifiable” putative caspase-3 DEVD↓X in the human proteome following digestion by trypsin with ArgC-like specificity (*red*) and LysN (*green*). *B*, LATE workflow for the comparison of two conditions. Extracted proteins of each sample are digested with LysN, and the resulting peptides undergo N-terminal-specific isotopic dimethylation. The samples are then mixed and subject to hydrophobic tagging of lysines, which allows for their removal and for the enrichment of the N-terminal peptides. The drawing was created with BioRender.com. *C*, LATE N-terminal dimethylation labeling specificity following LysN digestion. Over 95% of peptides’ N-terminal amines were labeled by dimethylation or naturally blocked by acetylation while less than 5% of the lysine side-chain amines were labeled. *D*, peptide-spectrum match (PSM) number of LysN internal peptides (*green*) *versus* the PSM number of ORF (*burgundy*) and neo N-terminal peptides (*blue*) before (PreLATE) and after LATE. *E*, comparison of human proteins with free (*left*) and acetylated (*right*) N-terminal peptides identified by HYTANE (*red*) and LATE (*green*). HYTANE, hydrophobic tagging-assisted N-termini enrichment; LATE, LysN amino terminal enrichment.

To improve the coverage of the N-terminome, we developed LysN Amino Terminal Enrichment (LATE), an N-terminal enrichment strategy based on LysN digestion and specific N-terminal α-amine isotopic labeling (Fig. 1*B*). We employed LATE in parallel to the HYTANE N-terminomics method to study the central cellular apoptosis protease, caspase-3. By applying this approach to a set of cell-based apoptosis experiments and controlled *in vitro* studies, we identified many reported as well as novel caspase-3 cleavages. With this comprehensive mapping of N-terminal peptides, we demonstrate how Nt-acetylation affects the ORF N-terminal susceptibility to cleavage by caspase-3 and find that caspase-3 cleavage can generate new sites for posttranslational N-terminal acetylation. We also show that these Nt-acetylation (neo-Nt-acetylation) events occur in the early stages of apoptosis. This is the first demonstration of a link between posttranslational neo-Nt-acetylation and caspase proteolytic pathways.

## Experimental Procedures

### Chemicals and Reagents

The following reagents were used in the study: LysN endoproteinase (Promega), sequencing-grade trypsin (Promega), sodium cyanoborohydride (Sigma-Aldrich), ^13^CD_2_-formaldehyde (Sigma-Aldrich), ^12^CH_2_-formaldehyde (Sigma-Aldrich), chloroacetamide (Sigma-Aldrich), glycine (Sigma-Aldrich), acetic acid (Sigma-Aldrich), undecanal (TCI), acetonitrile (JT Baker), formic acid (Biolabs), guanidinium-HCl (Spectrum), Hepes (Spectrum), ethanol (LiChorsolv), HPLC-grade water (Thermo), EDTA (Spectrum), acetic acid (Carlo Erba). All solvents used are MS grade.

### Plasmids

The construct for *Escherichia coli* expression of WT caspase-3 (pET23b-Casp3-His) was obtained from Addgene (plasmid 11821). The construct for mammalian expression of caspase-3/GFP was a gift from Dr Yaron Fuchs Lab (Technion).

### *E. coli* Proteome Preparation

*E. coli* DH5α cells were grown in LB medium until an absorbance of *A*_600nm_ = 0.5. Cells were harvested by centrifugation at 6000*g* for 10 min at 4 °C and washed twice with 50 mM PBS pH 7.4. The cell pellet was resuspended in 50 mM Hepes, 8 M guanidium-HCl pH 7.5 and lysed by heating at 95 °C for 10 min. The lysate was clarified by centrifugation at 15,000*g* for 10 min at 4 °C, and the supernatant was collected.

### Caspase-3 Expression and Purification

The expression construct of pET23b-Casp3-His6 was extracted from DH5α bacterial culture purchased from Addgene (Plasmid #11821). BL21-CodonPlus (DE3)-RIPL competent cells were transformed with the expression construct and plated on LB-agar supplemented with ampicillin (100 μg/ml) and chloramphenicol (34 μg/ml). A single colony of the transformants was inoculated into 5 ml LB supplemented with the above antibiotics and cultured at 37 °C and 200 rpm shaking, overnight. On the next morning, the overnight culture was inoculated into 50 ml LB supplemented with the above antibiotics and grown for 3 h at 37 °C and 200 rpm shaking. The culture was transferred to 1 l of LB in a baffled flask and cultured. At *A*_600nm_ = 0.6, the temperature was lowered to 17 °C and expression was induced by the addition of IPTG (1 mM). Expression was terminated after 16 h by harvesting the cells (centrifugation for 20 min at 4000*g* at 4°). The harvested cells were resuspended in 50 ml ice-cold buffer A (200 mM NaCl, 20 mM Tris-HCl, pH 7.5) and stored at −80 °C. The cells were lysed by three freeze-thaw cycles (liquid nitrogen and room temperature respectivley) followed by disruption of the cells using a cell disrupter (OS Model, Pressure Biosciences). The lysate was clarified by centrifugation (20,000*g*, 4 °C, 20 min), and the clear lysate was purified on HisTrap HP 1 ml affinity column (Cytiva) by FPLC system (NGC, Bio-Rad). The protein was eluted from the column using a linear gradient (elution buffer: 0–1 M imidazole, 200 mM NaCl, 20 mM Tris-HCl pH 7.5). The fractions were analyzed by SDS-PAGE. Based on SDS-PAGE, fractions containing caspase-3 were pooled together and dialyzed against 20 mM Hepes pH 7.5 and 80 mM NaCl. Aliquots of the protein were stored at −80 °C.

### Cell Culture and Transfection

Human colorectal carcinoma cells (HCT116) were grown in RPMI supplemented with 10% fetal calf serum (FCS), 2 mM L-glutamine, and 1% Pen-Strep solution. Immortalized aneuploid human keratinocyte cells (HaCaT) were grown in Dulbecco’s modified Eagle’s medium supplemented with 10% FCS, 2 mM L-glutamine, and 1% Pen-Strep solution, in 10-cm plates. When the cells reached 80% confluence, the medium was discarded and the cells were washed three times on the plate with 10 ml PBS before detachment or lysis.

For cell-based caspase-3 experiments, HCT116 cells were grown in a 10-cm plate until they reached 40% confluence, then the medium was changed to starvation medium (RPMI 2 mM L-glutamine, 1% Pen-Strep solution without serum) for 2 h. The transfection mix was prepared by mixing 15 μl Polyjet (SignaGen) with 6 μg of caspase-3/GFP bicistronic vector each prepared in 250 μl starvation medium; the mix was left for 10 min at room temperature before adding it to the starved cells. Eight hours following transfection the medium was changed back to RPMI supplemented with 10% FCS, 2 mM L-glutamine, 1% Pen-Strep solution, and GFP fluorescence was observed to estimate the efficiency. Next, the cells were split and grown to 80% confluence before sorting.

### Cell Viability Assay

Ten thousand HCT116 cells transfected with caspase-3/GFP bicistronic vector or nontransfected cells were seeded in 96-well plates and grown for 24 h in RPMI medium (as described above). Then the cells were treated (in triplicates) with different concentrations of BCL-2 inhibitor ABT-199 (0, 10, 50, 100, 150 μM). At each of the specified time points cell viability was measured using XTT-based Cell Proliferation Kit (Sartorius), according to the manufacturer’s protocol.

### Cell Lysis and Protein Extraction

For extraction under denaturative conditions, 1 ml of 95 °C preheated 50 mM Hepes, 8 M guanidium-HCl pH 8.0 was added to each plate, and cells were scraped down and incubated 5 min at 95 °C. Next, the cell lysates were sonicated using UP200St with VialTweeter (Hielscher) at max amplitude, 70% cycle time for 5 min. The sonicated lysates were centrifuged at 15,000*g*, 10 min, 4 °C, and the cleared supernatants were collected. For extraction under native conditions, cells were detached following 5 min of treatment with trypsin-EDTA and collected by centrifugation (5000*g*, 5 min, 4 °C). Cell pellets were washed three times with PBS and resuspended in 0.5 ml of 150 mM NaCl, 20 mM Hepes pH 7.5, and lysed by three freeze-thaw cycles of 5 min in liquid nitrogen followed by 20 min on ice. The lysate was clarified by centrifugation at 15,000*g* for 10 min at 4 °C. Protein concentration was determined by bicinchoninic acid assay (20).

### *In Vitro* Caspase Experiments

Clear lysate extracted under native conditions was split into two aliquots. One was left untreated (control) while the other was mixed with recombinant human caspase-3 in a 1:20 w/w ratio. Both samples were kept at 37 °C with gentle mixing. After 1, 6, and 18 h, a fraction of 200 μg protein was taken from the control and caspase-3-treated samples into a clean tube containing an equal volume of 20 mM Hepes, 8 M guanidium-HCl pH 7.5 and incubated at 95 °C for 5 min. For checking *in vitro* Nt-acetylation, the clear lysate was split into two aliquots. One was dialyzed by 3 cycles of concentration and 10× dilution at 4 °C in ice-cold 20 mM Hepes pH 7.8, 1 mM DTT, 1 mM EDTA, 100 mM NaCl using 3k cut-off Amicon Ultra 2 ml Centrifugal Filters (Merck). Equal amounts of protein from both dialyzed and undialyzed samples were treated with caspase-3 as described above. Aliquots were taken from each sample after 0.5, 6, and 18 h. Nt-acetylation samples were subject only to HYTANE.

### Cell-Based Caspase Experiments

HCT116 cells transfected with caspase-3/GFP vector were detached with trypsin-EDTA, quenched by an equal volume of medium, and centrifuged at 1500*g* for 5 min. Then the cells were resuspended in 1 ml RPMI supplemented with 10% FCS, 2 mM L-glutamine, 1% Pen-Strep solution and transferred to a 5-ml polystyrene round-bottom tube with a cell-strainer cap (Falcon). Unstained negative control cells were also prepared in the same way for population determination during sorting. Sorting was done using BD FACS-Aria (BD Bioscience) against GFP fluorescence. Between 1.5 and 2 million cells of GFP-positive (high caspase-3) and negative populations were collected per biological repeat. After sorting, samples were centrifuged at 1500*g* for 5 min, resuspended in 4 ml rich medium (RPMI, 20% FCS, 1% Pen-Strep, 2 mM L-glutamine), and seeded in a 6-cm plate. Cells were grown for another day while the medium was refreshed after 12 h. Lastly, samples were treated with ABT-199 at a final concentration of 150 μM to induce caspase-3 activation for 2 h, prior to harvesting the cells. For the time-course experiment of ABT-199, HCT116 cells were seeded in a six-well plate and grown until they reached 60% confluence. The cells were washed twice with fresh medium, and 2 ml medium with 150 μM ABT-199 or DMSO were added. After 0.5, 1, and 2 h, ABT-199- and DMSO-treated cells were washed three times with PBS, detached with trypsin-EDTA and centrifuged at 1500*g* for 5 min. The pellets were washed three times with PBS prior to lysis in 100 mM Hepes, 8 M guanidium-HCl pH 7.5.

### LysN Amino Terminal Enrichment

One hundred micrograms of protein was resuspended in 50 mM Hepes, 4 M guanidium-HCl pH 7.5 and reduced with 5 mM DTT at 65 °C for 30 min followed by alkylation with 12.5 mM chloroacetamide (CAA) for additional 20 min at room temperature in the dark. CAA leftovers were quenched with another dose of DTT, and the sample was diluted 1 to 4 with 20 mM Hepes pH 8 and the pH was further adjusted to 8.0 if needed using 1 N NaOH. Next, LysN (Promega) was added at 1:100 w/w and the samples were digested for 12 h at 37 °C. Digestion was terminated by adding a 6% final volume of glacial acetic acid. If required, additional acetic acid was added until the pH reached the range of 2.6 to 2.8. N-terminal α-amines–specific labeling was done by adding 40 mM (final) of C^13^D_2_ (heavy) or C^12^H_2_ (light) formaldehyde and 20 mM (final) of sodium cyanoborohydride at 37 °C for 10 min. Heavy and light labeled samples were quenched by addition of glycine (final concentration 100 mM) dissolved in 2% acetic acid and incubation at room temperature for 10 min. Next heavy- and light-labeled samples were mixed and desalted using OASIS-HLB 100 mg cartridge (Waters). Peptides were eluted with 60% acetonitrile and 0.1% formic acid and dried to completeness using CentriVap Benchtop Vacuum Concentrator (Labconco). Next, samples were resuspended in 100 μl of 100 mM Hepes pH 7.0. The hydrophobic modification was done by adding undecanal at 50:1 undecanal:peptide w/w ratio and 20mM final concentration of sodium cyanoborohydride. Undecanal was originally dissolved in ethanol to a final concentration of 20 mg/ml. The undecanal labeling was carried out at 50 °C for 2 h while having the sodium cyanoborohydride refreshed (∼40mM final concentration) after the first hour. Undecanal-labeled samples were centrifuged at 20,000*g* for 5 min at RT, and the supernatants were transferred to a new tube and dried to completeness using CentriVap Benchtop Vacuum Concentrator (Labconco). Dried samples were dissolved in 2% acetonitrile and 0.1% formic acid and desalted with OASIS-HLB 100 mg cartridge (Waters). Undecanal leftovers and undecanal-modified peptides should remain bound to the column after elution with 60% acetonitrile. Samples were dried and resuspended in 2% acetonitrile and 0.1% formic acid prior to LC-MS analysis.

### Hydrophobic Tagging-Assisted N-Termini Enrichment

One hundred micrograms of protein of each of the compared samples was resuspended in 50 mM Hepes, 4 M guanidium-HCl pH 7.5 and reduced with 5 mM DTT at 65 °C for 30 min followed by alkylation using 12.5 mM CAA for additional 20 min at room temperature in the dark. CAA leftovers were quenched with another dose of DTT. Each sample was labeled by adding 40 mM (final) of either C^13^D_2_ (heavy) or C^12^H_2_ (light) formaldehyde and 20 mM (final) of sodium cyanoborohydride and incubating at 37 °C overnight followed by quenching with 100 mM glycine (final) for 1 h at 37 °C. Next, heavy and light-labeled samples were mixed and diluted 1 to 4 using 20 mM Hepes pH 8.0. The mixed samples were digested with sequencing-grade trypsin (Promega) in a 1:100 w/w ratio, at 37 °C overnight. The tryptic digestion was quenched by the addition of formic acid to a final concentration of 1%. The combined sample was desalted using OASIS-HLP and dried completely in a SpeedVac. Undecanal-based enrichment was done as described for LATE.

### Liquid Chromatography–Mass Spectrometry

Desalted samples were subjected to LC-MS analysis using a Q Exactive Orbitrap Plus mass spectrometer Q Exactive Orbitrap HF coupled to Easy-nanoLC 1000 capillary HPLC except for time-course N-terminomics of HCT116 with ABT-199 that were done with Evosep One chromatography system. In the experiments done with Easy-nanoLC HPLC, the peptides were resolved by reversed phase using a homemade 30-cm-long 75-μm-diameter capillary, packed with 3.5-μm silica using ReproSil-Pur C18-AQ resin (Dr Maisch GmbH). Peptides were eluted using a linear 120-min gradient of 5 to 28% acetonitrile with 0.1% formic acid, followed by a 15-min gradient of 28 to 95% acetonitrile with 0.1% formic acid and a 15-min wash of 95% acetonitrile with 0.1% formic acid (at flow rates of 0.15 μl/min). In the experiment done with EvoSep One HPLC, the samples were loaded onto the EvoTip, followed by two washes with 20 μl buffer 0.1% formic acid. The washed peptides were kept wet by applying 150 μl of 0.1% formic acid on top of the EvoTip until submitted to MS. The loaded EvoTips were resolved on a 15 cm × 150 μm analytical column, packed with 1.9-μm C18 beads (EV1106). Peptides were separated over an 88-min gradient according to the manufacturer standard method. MS was performed in positive mode using an *m/z* range of 300 to 1800 in a data-dependent acquisition mode, a resolution of 70,000 for MS1, and a resolution of 17,500 for MS2, repetitively; full MS scans were followed by high-energy collisional dissociation of the 10 or 20 most dominant ions selected from the first MS scan for Q-Exactive Plus and Q-Exactive HF, respectively.

### Data Analysis

Data-dependent acquisition experiments were analyzed using Trans Proteomic Pipeline (v. 6.1) (21) based on COMET (v. 2020_01 rev 1) (22) searches. Time-course N-terminomics of HCT116 with ABT-199 were analyzed with Trans Proteomic Pipeline (version 6.2) and COMET (v. 2022_01 rev 1). Analyses of searches for acetylated, dimethylated, and methylated N termini were done separately using the search parameters listed in supplemental Table S11 and include mass tolerance for precursor and fragment ions of 20 ppm. Comet results were further validated by PeptideProphet (23) at a false discovery rate of peptide identification less than 1%. XPRESS was used for relative peptide quantification (24). Mass tolerance for quantification was 20 ppm with “The minimum number of chromatogram points needed for quantitation” set to 3 and the number of isotopic peaks to the sum set to 0. Searches were conducted against *Homo sapiens* proteome containing 73,248 sequences (ID: 9606, downloaded from UniProt in June 2019) and supplemented with cRAP sequences downloaded from ftp://ftp.thegpm.org/fasta/cRAP. Bioinformatic analysis of the N-terminome identifications was carried out using in-house Python scripts (available for download from the GitHub repository https://github.com/OKLAB2016/peptide-matcher and https://github.com/OKLAB2016/parse-and-unite) and based on UniProt annotations (25). These scripts were used to combine peptide identification into unified list, aggregate peptide ratios based on the sum of peak areas of different peptide-spectrum matches (PSMs) of the same peptide, map peptide location with the protein sequence, and provide secondary structure and relative solvent accessibility for the peptides and its flanking amino acids. Differential amino acid usage logos were created with dagLogo v. 1.32.0 (26). Logos were plotted as the percent difference of the amino acid frequency at each site against the background of the Swiss-Port human proteome. Boxplots were created using BoxPlotR (27). Gene ontology term enrichment analysis was performed with topGO v. 2.46.0 (28), and the results were visualized by plotting terms significantly enriched with respect to the whole human proteome (false discovery rate ≤ 0.01) in semantic coordinates obtained with rrvgo v. 1.6.0 (29).

### Experimental Design and Statistical Rationale

Cell-based proteomic analyses were done using triplicate cell cultures (biological replicates). Biological replicates were individual cultures grown separately and prepared separately. Once these cultures reached appropriate confluence levels, they were treated as described. *In vitro* proteomics analyses were done using a protein extract from one cell culture (one biological repeat). This extract was aliquoted, and each aliquot was subject to different treatments such as ±dialysis and/or ±incubation with caspase-3. These different treatments were sampled at different time points. The quantitative proteomics analysis was based on isotopic labeling. The abundance ratios of each sample (MS run) were normalized against the median ratio, and then quantitative data from identical peptide samples were aggregated together by summation. Significant ratio changes were defined based on a fixed threshold as described before (11). All data will be publicly available *via* respective repositories.

## Results

Most proteomics studies aimed at studying proteolytic processing and modification of protein N termini rely on negative selection methods. Many of these N-terminomics methods are based on blocking/labeling of lysine residues, which prevents the use of proteases with lysine cleavage specificity. Therefore, even though they utilize trypsin digestion, the resulting peptides are only those derived from cleavage at arginine residues (thus resembling ArgC digestion). This restriction limits the identification potential considerably; for example, identification of ORF N termini is possible for only less than 50% of the human proteome (supplemental Fig. S1). Although repeating the N-terminomics experiments with proteases other than trypsin can improve the coverage, there are still several thousand protein ORF N termini that can only be identified by a lysine-specific protease (supplemental Fig. S1). A major use of N-terminomics methods is for the identification of proteolytic cleavage sites. For example, in the human proteome, there are 134 putative cleavages of caspase-3, the apoptosis executioner protease whose canonical cleavage motif is DEVD↓X (30). Of these, only 73 (54%) can be identified by N-terminomics methods that are based only on cleavages at arginine residues. The number of identified caspase-3 cleavage sites can be considerably expanded to 106 (∼80%) by also using peptides generated from cleavage at lysine residues following LysN digestion (Fig. 1*A*). Similar expansion in the number of identifications was obtained when a less restrictive cleavage motif was used. Aside from the sequence coverage, it was also shown in previous studies that peptides with a basic residue closer to the N termini or the C termini have different chromatographic characteristics, which contributes to the peptide diversity between LysN and trypsin (ArgC-like) peptides (31). Based on these considerations, we developed LATE, a new method utilizing LysN digestion followed by specific N-terminal α-amines blocking while minimizing lysine modification, then exploiting the free lysine ε-amine to deplete internal peptides (Fig. 1*B*). After protein extraction and digestion, α-amines are labeled using reductive dimethylation in acidic buffer (32) and the compared samples are mixed. Internal peptides containing lysine residues are depleted by adding undecanal that reacts with lysine ε-amine, and this aids to separate them on a C18 column based on the added hydrophobicity (12, 16).

A key element in the efficiency of LATE is the specific dimethylation of peptides’ N termini (α-amines) without notable labeling of lysine residues (ε-amines). The conditions for this step were optimized following recent reports (32, 33). LATE labeling efficiency was tested on the pre-enriched sample where we observed labeling of free N-terminal α-amines with >95% efficiency, while only <5% of lysine side-chain amines were dimethylated (Fig. 1*C* and supplemental Table S1). Internal peptides that have Number of Enzymatic Termini = 2, those being full LysN peptides, dominated the pre-enrichment sample with more than 80% (Fig. 1*D*) accompanied by a low percentage of ORF N-terminal (*i.e.*, that begin at positions 1 or 2 of the predicted ORF) and neo-N-terminal peptides. Around 10-fold enrichment of ORF N-terminal and neo-N-terminal was achieved after applying LATE, with the incidence of internal peptides decreasing to less than 10%. The same enrichment trend was observed in our HYTANE sample (supplemental Fig. S2), similarly to the previous reports (12, 16). As predicted, application of LATE allowed identification of a significant number of dimethylated N-terminal peptides that were not identified by HYTANE (Fig. 1*E*). A similar trend albeit with a reduced magnitude was observed for peptides with acetylated N termini (Fig. 1*E*). Similar results were obtained when LATE and HYTANE were applied to the *E. coli* N-terminome (supplemental Fig. S3). In these two experiments LATE improved the overall coverage of the N-terminome, but the number of identified peptides with LATE was slightly lower than the number of peptides identified by HYTANE. We noticed that this reduction was more pronounced for acetylated N termini compared with dimethylated ones, as this may be due to the stronger hindrance of N-terminal amine ionization by the covalently bound acetyl group (34). We concluded that LATE is better suited for studying proteolysis rather than the natural acetylation of N-terminome, yet it provides valuable information and unique identifications on both.

**Fig. 2:**
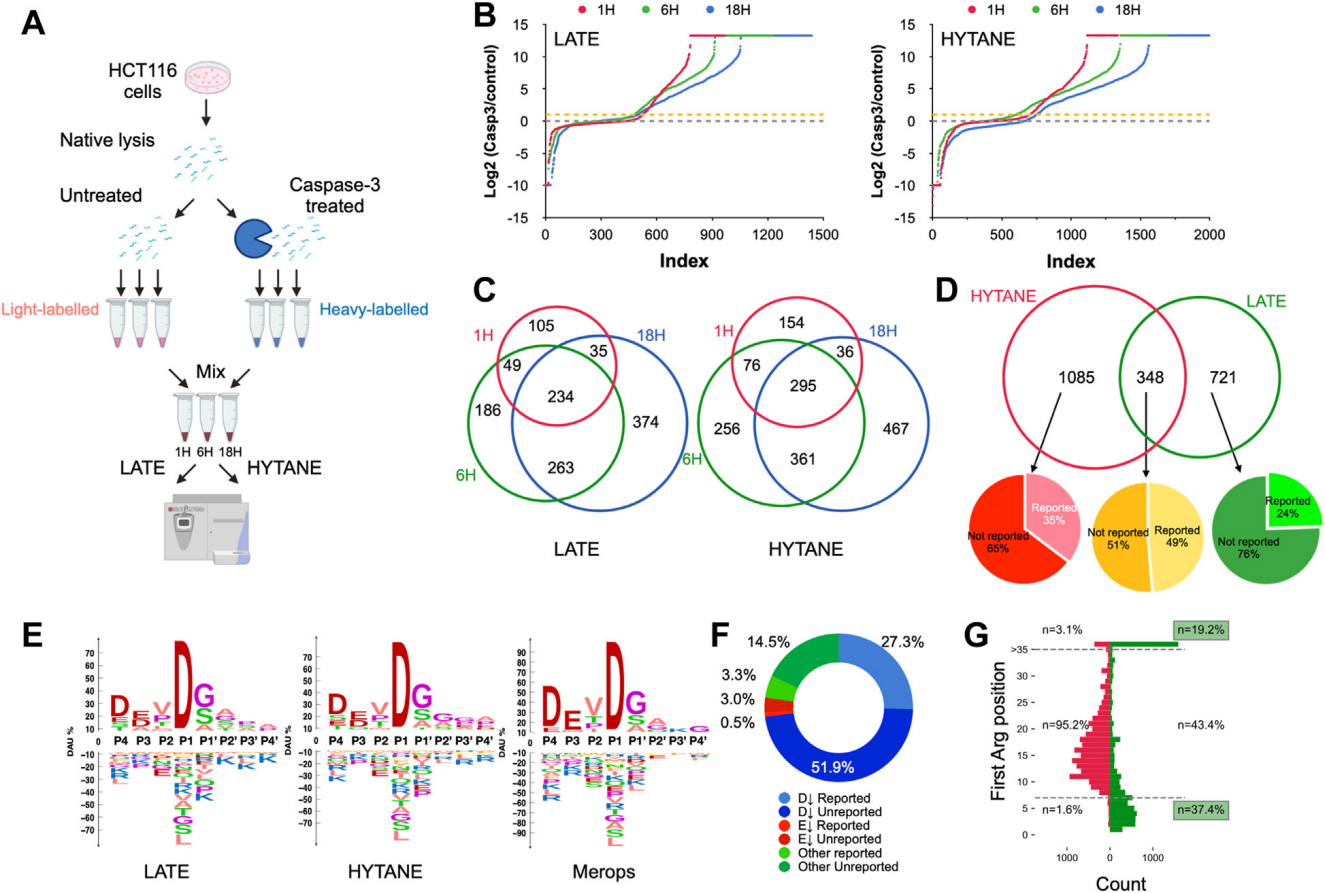
**N-terminomics of caspase-3 cleavages *in vitro*.***A*, *in vitro* experimental scheme. The drawing was created with BioRender.com. *B*, ranked ratios of dimethylated peptides showing the accumulation trends of peptides with high Log_2_ (caspase-3/control) abundance ratio (>1 as marked by the *yellow dotted line*) in LATE and HYTANE. *C*, Venn diagram of peptides with Log_2_ (caspase-3/control) >1 at each time point in LATE and in HYTANE. *D*, Venn diagram of the unique cleavage sites after D or E that were identified only in the caspase-3-treated samples with a caspase-3/control ratio ≥2 in at least one time point. “Reported/not reported” is based on a comparison with published data. *E*, sequence logo of all putative cleavage sites that were identified only in the caspase-3-treated samples with a caspase-3/control ratio ≥2, in comparison with all caspase-3 cleavage found in MEROPS. *F*, distribution of all putative cleavage sites that were identified only in the caspase-3-treated samples with a caspase-3/control ratio ≥2 based on the amino acid at the P1 position of their cleavage motif. *G*, the residue distance distribution of the nearest arginine to the identified peptide sequence in LATE (*green*) and HYTANE (*red*) experiments. HYTANE, hydrophobic tagging-assisted N-termini enrichment; LATE, LysN amino terminal enrichment.

Next, we applied LATE to study caspase-3 proteolytic activity *in vitro*. The intracellular proteome of HCT116 cells was extracted under native conditions and incubated with recombinant caspase-3 or buffer control at 37 °C. Equal aliquots were sampled at 1, 6, or 18 h of incubation and were subjected to N-terminal enrichment using LATE and HYTANE (Fig. 2*A*). Caspase-3-treated samples and control samples were labeled with heavy and light dimethylation respectively. Successful enrichment of the N-terminome was obtained with both LATE and HYTANE (supplemental Fig. S4). Peptides with a high relative abundance ratio (caspase-3/control) are putative products of caspase-3 cleavage. Yet, since caspase-3 might activate other proteases (including other caspases), not all putative cleavages are necessarily direct caspase-3 products. The distribution of the caspase-3/control abundance ratio in the log_2_ scale was centered around zero (*i.e.*, no change), with the frequency of peptides with a high caspase-3/control abundance ratio increasing over time in both methods (Fig. 2*B*). To distinguish the background proteolysis from the caspase-3 cleaved proteins, we considered only peptides that had a caspase-3/control ratio higher than 2 (Log_2_ of 1) as an indication for putative caspase-3 cleavages. Following this, we identified over 900 different cleavage sites after 18 h of incubation in each one of the methods with more than 200 sites identified at all time points (Fig. 2*C*, supplemental Tables S2 and S3). Caspase-3-mediated proteolysis has been studied extensively and its specificity is well defined (30, 35, 36). The sequence logo of the eight amino acids (P4-P4') flanking the reported caspase-3 cleavages in the MEROPS peptidase database (37) shows a very similar motif to the logos obtained from our data (Fig. 2*E*). By including only cleavages with a caspase-3/control ratio of ≥2 that were identified only in the caspase-3 treated samples and occurred after Asp or Glu, we selected the most credible caspase-3 cleavage sites. This way, we identified 721 additional putative caspase-3 cleavage sites with LATE that could not be identified with HYTANE (Fig. 2*D*). By combining the results of both methods, we achieved an ∼50% increase in the total number of putative cleavages obtained by HYATNE alone, reaching a total of 2154 putative cleavages enriched in the caspase-3-treated samples.

Of these putative cleavages, 733 (∼34%) matched previously reported caspase-3 cleavages in TopFind (38), CASBAH (36), DegraBase (39), MEROPS (37), and a recent caspase-3 N-terminomics study (40), while 1421 (∼66%) have not been reported so far. Some of these unreported cleavages occurred in 372 proteins that were not known previously as caspase-3 substrates (Fig. 2*F* and supplemental Tables S2 and S3). Interestingly, the proportion of unreported/novel putative caspase-3 cleavages obtained with LATE was higher than with HYTANE (74% *versus* 63%, respectively) (Fig. 2*D*, supplemental Tables S2 and S3). We were also able to identify 91 additional putative caspase-3 cleavage sites with Glu at P1 (supplemental Tables S2 and S3), consistent with caspase-3 ability to cleave after Glu residues (41). To evaluate the additional contribution of LATE to the results that can be achieved by ArgC-based N-terminomics methods like HYTANE, we compared the sequences of 7 to 35 amino acids downstream of the identified cleavage sites by each method. HYTANE requires an arginine at one of these 7- to 35-amino acid segments while LATE requires a lysine at these positions. As shown in Figure 2*G*, more than 50% of LATE identifications consisted of sequences containing Arg residues outside positions 7 to 35 downstream of the cleavage site and a similar trend was found in the location of Lys residues in the peptide identified by each method (supplemental Fig. S5). This demonstrates LATE’s ability to identify cleavage sites that cannot be found with HYTANE and vice versa.

**Fig. 3:**
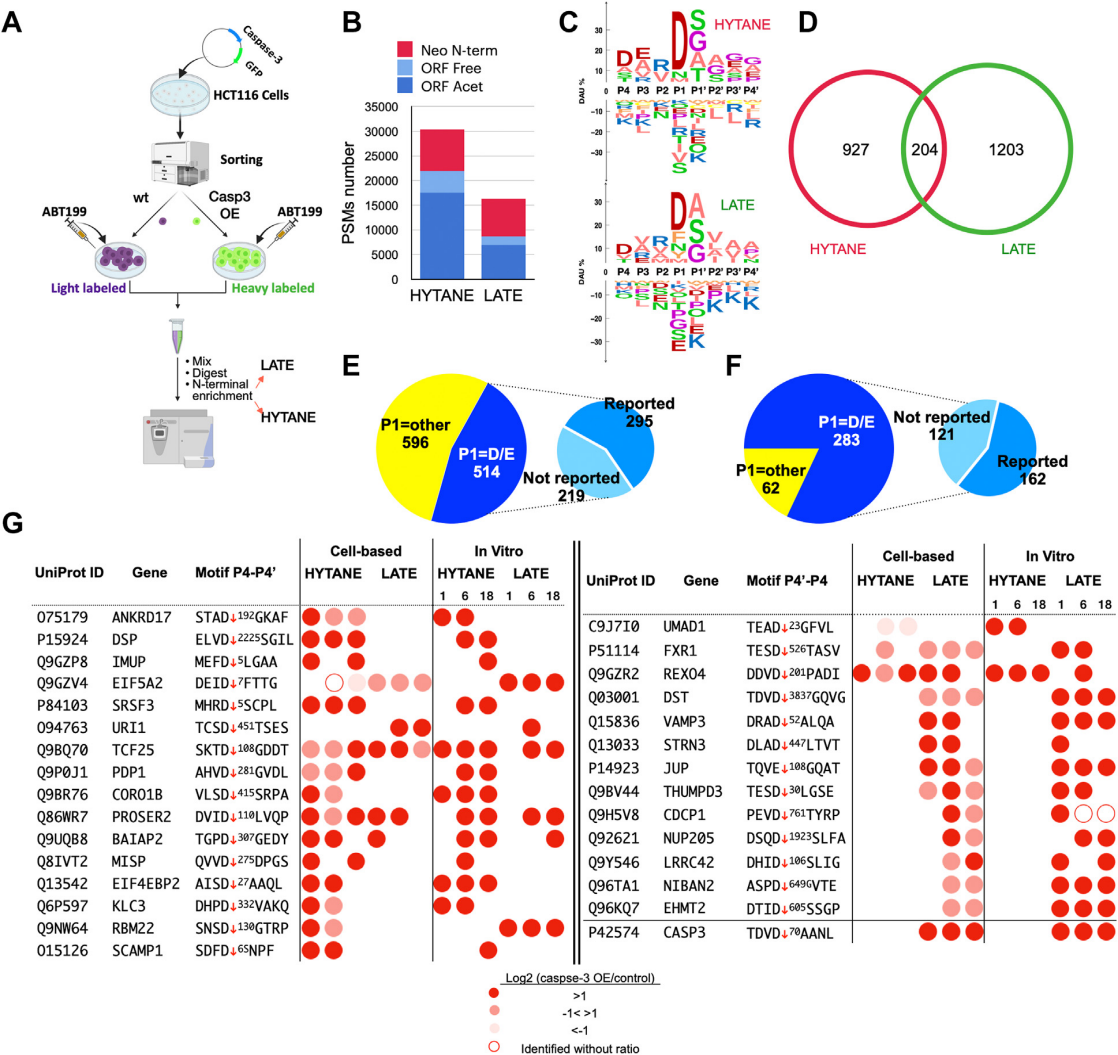
**N-terminomics of caspase-3 cleavages in cells.***A*, cell-based experimental scheme to study caspase-3-mediated cleavages in HCT116 following treatment with ABT-199. The drawing was created with BioRender.com. *B*, number of N-terminal peptides identifications following HYTANE and LATE enrichments. *C*, sequence logo of neo-N-terminal peptides cleavage motifs by HYTANE (*top*) and LATE (*bottom*). *D*, Venn diagram of the combined number of neo cleavage motifs identified by HYTANE (*red*) and LATE (*green*). *E*, the number of putative caspase cleavage motifs (with P1 = D or E) out of the total neo-N-terminal cleavage motifs that were identified. The categorization of reported/not reported is based on a comparison with published data (TopFind, etc.). *F*, the number of putative caspase cleavage motifs (with P1 = D or E) that had a high caspase-3/control abundance ratio (Log_2_ ≥ 1) out of the total neo-N-terminal cleavages identified. *G*, novel caspase-3 substrates and their cleavage sites. The cleavage site (caspase-3/control) abundance ratio is indicated by color. HYTANE, hydrophobic tagging-assisted N-termini enrichment; LATE, LysN amino terminal enrichment.

Next, we used the same combined N-terminomics approach to study caspase-3 substrates in cells. To this end, HCT116 cells were transfected with a bicistronic caspase-3/GFP plasmid and sorted using fluorescence-activated cell sorting to generate caspase-3-overexpressing cells (GFP-positive) and nontransfected cells (no GFP). Caspase-3 activity was induced by ABT-199 in both populations (Fig. 3*A*). Overexpression of caspase-3 accelerated apoptosis in HCT116 compared with nontransfected cells in response to ABT-199 (supplemental Fig. S6*B*). We used LATE and HYTANE to characterize the ORF and neo-N-terminal peptides in those cells. This way, changes in proteolytic processing between these cell populations could be directly attributed to caspase-3. The majority of identified peptides were ORF N-terminal peptides, and most of those were acetylated (Fig. 3*B*) with ∼57% of the total identifications in HYTANE and ∼43% in LATE. Yet, the number of neo-N-terminal peptides identified was similar in both methods (Fig. 3*B*) and the sequence logos of those peptides were similar to each other with strong dominance of Asp at the P1 position (Fig. 3*C*). Both logos were also similar to those we obtained from the caspase-3 *in vitro* approach and from caspase-3 cleavages based on MEROPS (Fig. 2*E*). This indicates the dominance of caspase-3 processing among the neo-N-terminal peptides. Excluding the N-terminal peptides of ORFs or alternative translation initiation sites and N-terminal peptides resulting from known protein processing events, such as signal peptide or prodomain removals, we detected 2330 neocleavage sites for 1117 different proteins. Although with LATE fewer overall PSMs were obtained than with HYTANE (Fig. 3*B*), it enabled the identification of more neo-N-terminal peptides (1407) than HYTANE (1127) (Fig. 3*D*, supplemental Tables S4 and S5).

To chart the changes in the N-terminome we combined the identifications obtained by both methods and considered only those that were identified at least twice. Altogether, 1127 neocleavage sites were identified, and from these, 519 (∼46%) were cleavages after Asp or Glu corresponding to the caspase family specificity (Fig. 3*E*). Of these putative caspase cleavages, 221 (∼42%) were at sites that were not reported so far in proteomic studies of caspases (Fig. 3*E*, supplemental Tables S4 and S5). Some of these unreported cleavages occurred in 65 proteins that were not known so far as caspase-3 substrates (supplemental Table S6). A closer look at the Log_2_ (caspase-3/control) abundance ratios of the 298 caspase cleavage sites previously reported in other proteomics studies shows that the majority are larger than 1 and a significant number of the Log_2_ ratios are within the range of −1 to 1 (Supplemental Fig. S7). Caspase-3 was activated by ABT-199 (42) in both the caspase-3 overexpressing and control cells; hence, there is expected to be no or only a small difference between the two treatments for substrates that are cleaved effectively by the endogenous caspase-3. Therefore, in this work, we also report cleavages without large fold change as putative caspase-3 cleavages. Of note, despite the difference in background proteolysis between the cell-based (Fig. 3*A*) and the *in vitro* experiments, when we compared the abundance ratios of the same substrate cleavage across the two experiments, they showed similar trends (supplemental Fig. S8). Moreover, for substrate winnowing of caspase-3-related cleavages, we filtered the data further and considered only cleavages that show at least a 2-fold increase in the cells that overexpressed caspase-3 (Fig. 3*F*). We identified several putative caspase-3 cleavages that have not been reported previously, either in the various studies related to apoptosis or in caspase-3-related proteomics studies (36, 38, 39, 40) (Fig. 3, *G* and *F* and supplemental Table S6). Of the 345 cleavages that show significant change, 283 (over 80%) were with caspase specificity. Of these, 162 (57%) were at sites that were previously reported and 121 (43%) at sites that were not reported. From these unreported 121 sites we selected several substrates and demonstrated that they are cleaved by caspase-3 *in vitro* (Fig. 3*G*, supplemental Tables S2 and S3). All the novel substrates and their cleavage sites are shown in supplemental Table S6. Of note, one of the unreported cleavage sites identified in all of our experiments, exclusively by LATE, is a cleavage site at position D70 of caspase-3 itself (Fig. 3*G*).

We used gene ontology enrichment analysis to compare the list of unreported putative caspase-3 substrates that we identified with previously reported substrates from apoptosis and caspase-related proteomic studies. The new putative substrates show a pattern highly similar to the proteins previously reported to be cleaved by caspase-3, thus demonstrating that the new cleavages come from substrates with similar biological function to the already known ones (supplemental Fig. S9). The number of potential caspase-3 cleavages and substrates identified in the cell-based experiments was smaller than that obtained in the *in vitro* experiments. In order to understand whether there is a structural basis for the differences between the cleavages obtained in the cell-based and *in vitro* experiments, we compared the secondary structure contexts and solvent accessibility around the cleavage sites. This comparison revealed very similar secondary structure contexts for the two experiments (supplemental Fig. S10*A*) but a significant difference in the relative solvent accessibility (supplemental Fig. S10*B*), with the cleavages identified in cells occurring in more exposed regions than those obtained *in vitro*. This might reflect the impact of cell lysis in the *in vitro* experiments that alters some protein structures and disrupts cellular compartmentalization that is kept under control in the cell-based experiments.

**Fig. 4:**
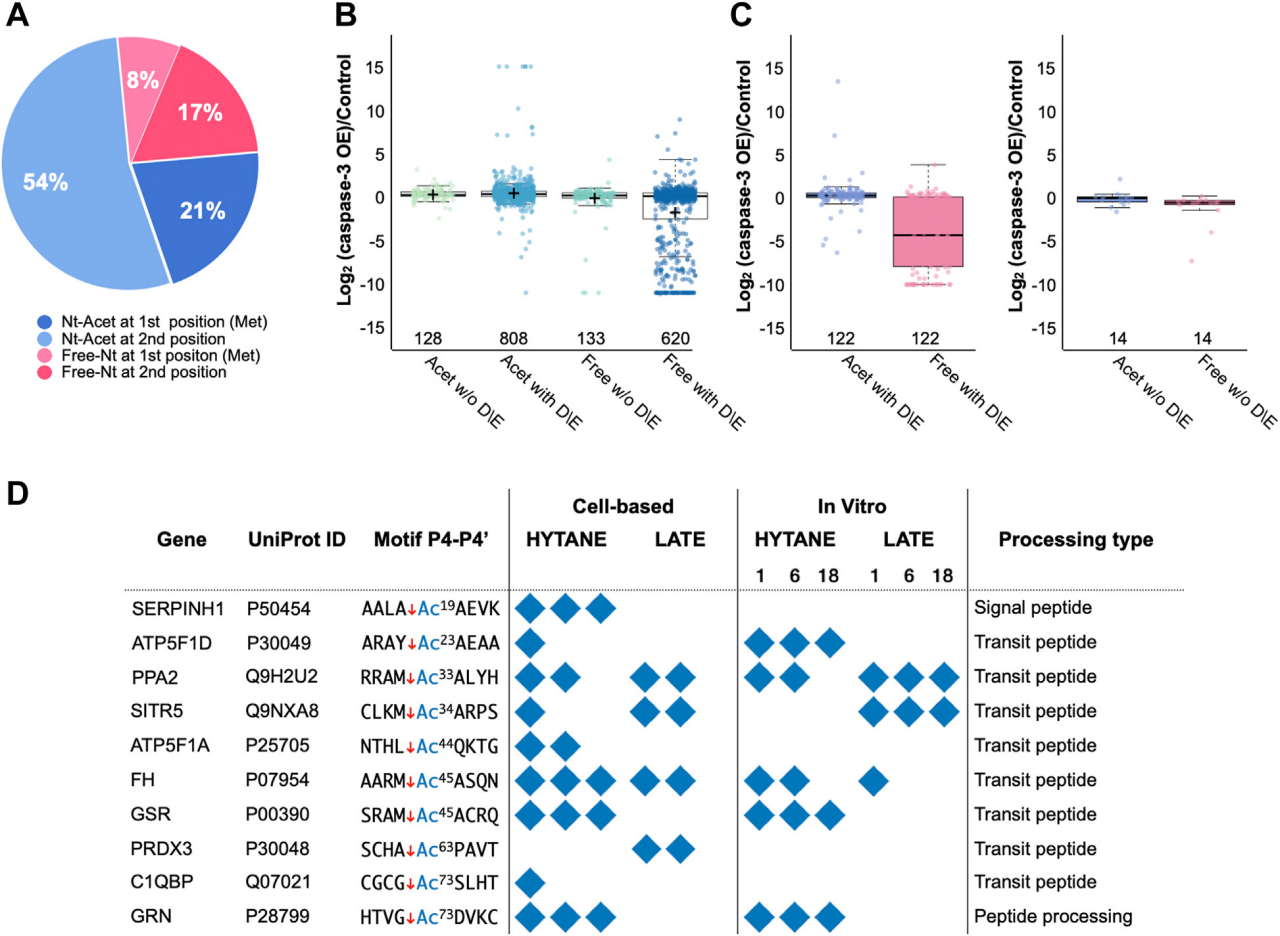
**Acetylation of both ORF and neo N-terminal peptides.***A*, the ORF N-terminal peptides identified in HCT116 cells overexpressing caspase-3 and their control were categorized based on the presence of initiation methionine (marked as first Met) or its removal (marked as second position) and the presence of Nt-acetylation (“Nt-Acet”; in shades of *blue*) or its absence (“Free”; in shades of *pink*). *B*, the abundance ratio distribution of the ORF N-terminal peptides in cells overexpressing caspase-3 and their control, based on their N-terminal state (free/acetylated) and the presence of Asp or Glu residue in their sequence. Box limits indicate the 25th and 75th percentiles; whiskers extend 1.5 times the interquartile range from the 25th and 75th percentiles; *crosses* represent sample means; data points are plotted as *open circles*. n = 128, 808, 133, 620 sample points. *C*, the abundance ratio distribution of the 122 ORF N-terminal peptides that were identified both as Nt-acetylated and with free N terminus and contain Asp or Glu in their sequence. *Center lines* show the medians; box limits indicate the 25th and 75th percentiles; whiskers extend 1.5 times the interquartile range from the 25th and 75th percentiles; data points are plotted as *open circles*. *D*, posttranslational neo-Nt-acetylation sites that were identified at N termini generated following known proteolytic processing of precursor proteins as indicated in UniProt. The *blue diamonds* represent identification in biological repeats or time points. HYTANE, hydrophobic tagging-assisted N-termini enrichment; LATE, LysN amino terminal enrichment.

One of the advantages of the negative selection N-terminomics methods is that in addition to the enrichment of the neo-N-terminal peptides, these methods also allow the characterization of the ORF N-terminal peptides (Fig. 1, *E* and *F*) (11). We used the same approach applied to the neo-N-termini peptides and combined the ORF N-terminal peptides identification obtained by LATE and HYTANE. In our search we included these known modifications of protein N termini: removal of the initiator methionine (17, 43) and Nt-acetylation (19). Together, the ORF N-terminal peptides of 2349 proteins were identified (supplemental Table S7). Similarly to published data (44), 74% of the peptides were Nt-acetylated, 26% were with free N termini (Nt-free), and 70% of all peptides underwent methionine excision regardless of their Nt-acetylation status (Fig. 4*A*). In addition, the frequencies of the first and second amino acids in Nt-acetylated and Nt-free ORF peptides (supplemental Fig. S11) were similar to those found in other proteomic studies (44).

Next, we checked if the nature of the ORF N-terminal modification affects the susceptibility of ORF N termini to cleavage by caspase-3. As can be seen in Figure 4*B* and supplemental Fig. S12, the abundance ratio of Nt-acetylated ORF peptides was distributed around zero regardless of the presence of Asp or Glu in their sequence. A similar trend was seen for Nt-free ORF peptides that do not contain potential caspase-3 cleavage sites. However, Nt-free ORF peptides with Asp or Glu in their sequence were more susceptible to cleavage by caspase-3 and their ratios deviated from a normal distribution as many of them have demonstrated lower abundance ratios in caspase-3-overexpressing cells relative to the control cells. Among these ORF N-terminal peptides, 136 were identified and quantified both in the Nt-acetylated form and in the free N-terminal form. Of these peptides, 122 contained Asp or Glu residues, thus containing putative caspase cleavage motifs. The abundance ratios of these peptides in their N-terminal acetylated form were spread around zero (no change) in log(caspase-3/control) scale while the abundance ratios of their free N-terminal form were much higher in the control (Fig. 4*C*). The abundance ratio of the 14 peptides that did not contain Asp or Glu in their sequence was distributed around zero regardless of the nature of their N terminus (supplemental Fig. S13). This supports the notion that Nt-acetylation may have a protective and stabilizing role as has been suggested in some studies (45). Nt-acetylation is defined mostly as a cotranslational modification, but there are a few examples of posttranslational Nt-acetylation (46, 47, 48, 49, 50). To investigate the presence of posttranslational Nt-acetylation that is not at the protein’s ORF N terminus, we checked the N-terminal peptides that were generated following known proteolytic processing of proteins. These processing events include signal/transit peptide or propeptide removal and peptide or precursor protein chain cleavages that are part of protein maturation or activation. By combining LATE and HYTANE results, we identified 329 posttranslational proteolytic processing sites reported in UniProt for 262 proteins (supplemental Table S8). Of these, 10 neo-Nt-peptides from 10 proteins were found with Nt-acetylation (Fig. 4*D*) as well as with free N terminus. The neo-acetylated-Nt-peptides identified were mainly from the mitochondrial proteins and derived from the removal of the mitochondrial transit peptide. This type of proteolytic processing is done after the protein is imported into the mitochondria and reaches the mitochondrial matrix (51) or inner membrane (52). Next, we looked for additional hints of posttranslational neo-Nt-acetylation that occur following proteolytic cleavages not annotated in UniProt (25) and not reported as an alternative initiation site in TopFind (38).

**Fig. 5:**
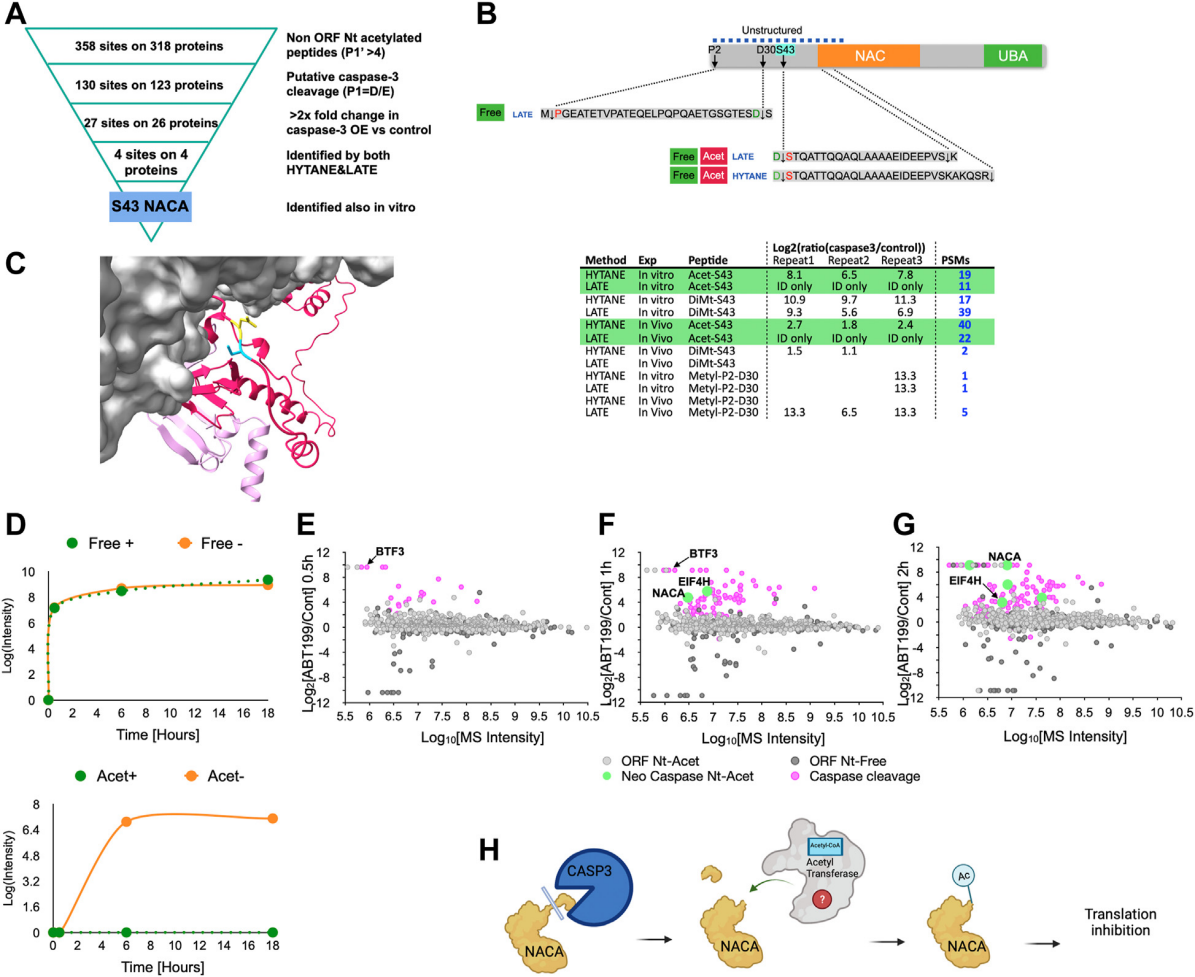
**Neo-Nt-acetylation of nascent polypeptide–associated complex A (NACA) of ORF and neo-N-terminal peptides.***A*, winnowing of acetylated neo-N-terminal peptides identified in HCT116 cells. *B*, the N-terminal peptides of nascent polypeptide–associated complex subunit alpha (NACA) identified in HCT116 cells. Cleaved peptide sequences and modifications are shown on the top and the peptide-spectrum match (PSM) numbers in the different experiments are on the *bottom*. *C*, the position of NACA cleavage (in *yellow* and *light blue*) by caspase-3, before Ser43, lead to the formation of a neo-Nt-acetylated form of NACA. The structural model is based on the structure of the ribosome-nascent chain containing an endoplasmic reticulum signal sequence in a complex with the nascent polypeptide–associated complex (Protein Data Bank: 7QWR). The ribosome and RNA are in gray. The full NACA (in *dark pink*) and BTF3 (in *light pink*) structures are based on AlphaFold (86) prediction and were aligned on top of the original partial structures. *D*, time-course of *in vitro* NACA cleavage and Nt-acetylation. The *upper panel* shows NACA cleavage at Ser43 by caspase-3 with (*green*) or without (*orange*) lysate dialysis before caspase-3 addition. The *lower panel* shows NACA neo-acetylation at Ser43 after caspase-3 cleavage with (*green*) or without (*orange*) lysate dialysis before caspase-3 addition. *E, F* and *G*, time-course N-terminomics of HCT116 cells treated with ABT-199 or DMSO (control). Log_2_ abundance ratios for individual peptides are plotted on the *y*-axis, and the corresponding total peptide mass spectrometry (MS) intensities are shown on the *x*-axis. The *circle colors* correspond to the peptide type: ORF Nt-Acetylated (*light gray*), ORF peptides with Nt-free (*dark gray*), caspase-generated neo-Nt peptides (*pink*), and neo-Nt-acetylated peptides (*light green*). *H*, the suggested order of events for NACA neo-Nt-acetylation following caspase-3 processing. HYTANE, hydrophobic tagging-assisted N-termini enrichment; LATE, LysN amino terminal enrichment.

We found 358 such neo-Nt-acetylation motifs in 318 different proteins (supplemental Table S9). To check if there were neo-Nt-acetylation sites in the list that were dependent on caspase-3 cleavage, we used the winnowing workflow shown in Figure 5*A*.

Consequently, we focused on the proteolytic cleavage and neo-Nt-acetylation of nascent polypeptide–associated complex A (NACA). The tandem mass spectrometry identifications of this neo-Nt-acetylated peptide by LATE and HYTANE had high scores and high peptide sequence coverage and provided conclusive evidence for the presence of Nt-acetylation on Ser43 (supplemental Fig. S13). NACA is a subunit of the heterodimeric ribosome-associated (53) nascent polypeptide–associated complex (NAC). The NAC complex is highly conserved among eukaryotes, and when associated with the ribosome it interacts with nascent polypeptide chains and mediates their sorting to different cellular compartments (54). In addition to the neo-Nt-acetylation of the peptide starting at Ser43, we found several neo-N-terminal peptides of NACA (Fig. 5*B* upper part). The majority of these cleavages were also reported in other apoptosis-related studies (36, 38, 39). Interestingly, in the cell-based experiments, this peptide was identified almost exclusively in its acetylated form with a total of 62 PSMs *versus* 2 PSMs for the free form, while in the *in vitro* experiments the total PSM numbers of both forms were similar. The location of this cleavage is within the N-terminal unstructured region of NACA, which makes it suitable for caspase cleavage. Based on a recent structure of the NAC complex bound to the ribosome (54) this region remains accessible even when NACA is in complex with BTF3 (nascent polypeptide–associated complex B, NACB) and the ribosome (Fig. 5*C*). The NACA Nt-acetylated Ser43 peptide was identified *in vitro* only after the addition of active caspase-3, which allowed us to study its generation process. We used HCT116 cell lysate, and half of it was subjected to dialysis to reduce acetyl-CoA concentration and prevent the acetylation reaction. Following the addition of active caspase-3, aliquots from both dialyzed and nondialyzed lysates were sampled at different time points and subjected to N-terminal enrichment and MS analysis. As shown in Figure 5*D*, the dialysis did not affect caspase-3 cleavage and the appearance of NACA neo-Nt-Ser43. This peptide was already identified 30 min after caspase-3 addition and was generated at an identical rate and intensity in both samples. The NACA neo-Nt-acetylated Ser43 peptide appeared at a later time point (6 h) but only in the nondialyzed sample (Fig. 5*E*). Next, we checked if and at which stage NACA neo-Nt-acetylation can be observed during apoptosis. HCT116 cells were treated with ABT-199 and DMSO (control). Cells were collected from the ABT-treated and control cells after 0.5, 1, and 2 h after addition of ABT-199 or DMSO and were then subjected to N-terminal peptide enrichment using HYTANE. No cell death was observed until 2 h of treatment (supplemental Fig. S6*B*). As shown in Figure 5, *E*–*G*, NACA neo-Nt-acetylation at Ser43 was already observed after 1 h of treatment (marked in green). Besides NACA, neo-Nt-acetylation was also identified on the translation elongation factor eIF4H after it was cleaved by caspase (at Ser94). Interestingly, we also observed a cleavage (occurred after 0.5 h of treatment) of BTF3, the protein that forms the ribosome-adjacent NAC with NACA (54, 55). Together with the rest of our results, this, for the first time, reveals the presence of posttranslational Nt-acetylation that is directly linked to caspase-3 proteolytic processing (Fig. 5*H*).

## Discussion

The use of multiple proteolytic enzymes in bottom-up proteomics studies is known to improve the number of protein identification and sequence coverage (56, 57). Similar improvements were also shown in several terminomics studies that combined trypsin with other proteases while using the same terminal peptides enrichment methodology (58, 59). Here we describe LATE, a simple methodology that is based on digestion with LysN for N-terminome characterization. LysN digestion has been used for the identification of ORF Nt-acetylated peptides (60, 61) but not for free ORF N-terminal peptides or neo-N-terminal peptides generated by proteolysis. A key element of LATE is the N-terminal-specific labeling by dimethylation, which also allows the use of isotopic labels and quantitative analysis. Unlike the few other reported N-terminal-specific labeling reagents, like TMPP (62) or 2-pyridinecarboxyaldehydes (14, 63), the required reagents are inexpensive and the labeling efficiency is very high and consistent. We demonstrated the usefulness of LATE combined with HYTANE in the investigation of caspase-3-mediated cleavages during apoptosis. This subject has been extensively studied, particularly by terminomics methodologies (13, 41, 64, 65, 66). We identified many novel caspase-3 cleavage sites and substrates as well as some known substrates whose cleavage by caspase-3 had not been previously identified by proteomic analysis. Such known substrates are desmoplakin (DSP) and plakoglobin (JUP), which have been known as caspase-3 targets for over 2 decades (67, 68) but their cleavage sites had not yet been identified by proteomics. We identified DSP and JUP cleavage sites as part of a large-scale study for the first time. The novel substrate list includes several proteins that are understudied in the context of apoptosis, for example, the unconventional prefoldin RPB5 interactor 1 (URI1), which is important to DNA stability (69) and affects apoptosis upon its phosphorylation (70), and Transcription factor 25 (TCF25 or NULP1), which binds the apoptosis inhibitor XIAP (71) and the overexpression of which induces cell death. Our combined system-wide *in vitro* and cell-based experiments allowed us to validate these proteins as caspase-3 substrates. In addition, this thorough N-terminomics characterization enabled us to monitor not just proteolytic cleavage but also changes in protein ORF N termini. In this respect, our results indicate that Nt-acetylation protects proteins' N-terminal regions from proteolytic cleavage by caspase-3 during apoptosis. In mammalian proteins, Nt-acetylation can be seen in more than 70% of ORF N termini, yet its physiological functions are not entirely understood. Nt-acetylation affects the charge of proteins' N termini; hence, it would be expected to modify their function. Initially, it was suggested that a general function for Nt-acetylation is the protection of proteins from proteolytic degradation (72, 73). Later on, it was suggested that, under certain conditions, Nt-acetylation might also be a degradation signal (degron) that marks the protein for ubiquitinylation and its destruction by the proteasome (74). We showed that, in the context of apoptosis, the presence of Nt-acetylation has a protective effect and prevents cleavage around the protein N-terminal by caspase-3.

Most Nt-acetylation events are thought to be cotranslational, but several posttranslational Nt-acetylation events were reported too. Among them are the Nt-acetylation of secreted proteins in Apicomplexa (75), cleaved proteins in yeast by an unknown protease (48, 49), transmembrane proteins in the Golgi (46), and cleaved actin (47). Nt-acetylation of nuclear-coded proteins imported to the mitochondria has been described, but only cotranslational ORF Nt-acetylations were identified (76, 77). Here we uncover the Nt-acetylation of several mitochondrial proteins that occur after their transit peptide removal in the mitochondrial matrix. Nt-acetylation of imported nuclear-encoded proteins after the cleavage of their transit peptides was identified in chloroplasts (77, 78, 79). Both organelles are thought to have a prokaryotic origin, yet Nt-acetylation is rare in bacteria (77). This raises the question of which N-acetyltransferase catalyzes the acetylation of mitochondrial proteins. Recently, a family of dual lysine and N-terminal acetyltransferases was identified and several of its members were shown to reside within plastids (80). Mammalian mitochondrial N-acetyltransferase has not been identified.

We also revealed several caspase-3-generated neo-N-termini that undergo posttranslational Nt-acetylation. We focused on and validated the caspase-3 dependence of NACA neo-Nt-acetylation that was dominant in all of our experiments and analyses. Aside from NACA's role as part of a ribosome-associated complex, it was shown to function as a transcriptional coactivator regulating bone development and hematopoiesis. Hence, its cleavage and Nt-acetylation might have implications on transcription. As part of a ribosome-associated complex, NACA binds to nascent proteins that lack a signal peptide motif as they emerge from the ribosome, while blocking their interaction with the signal recognition particle and preventing mistranslocation to the endoplasmic reticulum (54). Downregulation of NACA, by hypoxia or RNAi, can lead to the initiation of an endoplasmic reticulum stress response that is followed by caspase-dependent apoptosis (81, 82). The N-terminal region of NACA cleaved by caspase-3 contains a cluster of negatively charged residues (supplemental Fig. S14 top). This negatively charged region was shown to downregulate ribosome binding of NAC (55). It was suggested that this negative N-terminal region may interact with the positively charged ribosome-binding motifs of both NACA and its partner BTF3. Deletion of the N-terminal region increased NACA binding to the ribosome and caused severe translation inhibition (55). Therefore, the apoptosis-induced caspase cleavage of NACA is expected to have a similar inhibitory effect on translation. Based on the Nt-acetylation's stabilizing effect on protein termini, these observations suggest that neo-Nt-acetylation protects the malfunctioning NACA and thus promotes translation inhibition. Interestingly, we also observed a cleavage of BTF3 (Fig. 5, *E* and *F*), which appeared at the earliest time point. Similarly to the cleavage of NACA at Ser43, BTF3 cleavage after Asp175 led to the removal of a cluster of negatively charged residues (supplemental Fig. S14 bottom). Thus, it is likely that caspase-3 cleavage of BTF3 also increases NAC binding to the ribosome and likewise inhibits translation. We also observed neo-Nt-acetylation following caspase cleavage of the eukaryotic translation initiation factor 4H (eIF4H) (Fig. 5, *E* and *F* and supplemental Table S10). This protein was shown to enhance the activity of the initiation factor complex eIF4F by stimulating the helicase activity of eIF4A (83). The cleavage of eIF4H by caspase-3 between Asp93 and Ser94 lies directly in the middle of its RNA recognition motif and therefore is expected to abolish its activity and inhibit the RNA priming activity of the whole eIF4F. The neo-Nt-acetylation of eIF4H may shield the cleaved protein from additional proteolytic processing, thus maintaining the inhibition of RNA priming and of translation initiation. We also identified caspase-mediated cleavages of several factors that control translation elongation like eIF5A and eIF5A2, as has also been reported before (84). Some of these cleavages were observed shortly after apoptosis induction (supplemental Table S10). This demonstrates that translation inhibition begins during the early phases of apoptosis and that it is achieved by the combination of ribosome blocking, interference with translation initiation, and elongation blocking.

Together, our data reveal an interplay between caspase-mediated proteolysis and posttranslational Nt-acetylation. A similar functional interplay was described before for caspase-mediated proteolysis and phosphorylation (41, 85). It was shown that caspase cleavage can expose new sites for phosphorylation and that phosphorylation sites can enhance caspase cleavage in their vicinity (85). We found that caspase-3 cleavage can unveil new sites for posttranslational Nt-acetylation and conversely that Nt-acetylation protects proteins from caspase cleavage. It would be interesting to combine N-terminomics using LATE and HYTANE with phosphoproteomics to investigate if there is tripartite cross talk between caspase-mediated proteolysis, phosphorylation, and Nt-acetylation and if these modifications outcomes are additive or antagonistic to each other.

## Data Availability

The data that support the findings of this study have been deposited to the ProteomeXchange Consortium (http://proteomecentral.proteomexchange.org) *via* the PRIDE partner repository with the dataset identifiers: *In vitro* caspase-3 cleavages: PXD036648; in cells caspase-3 cleavages: PXD036593; apoptosis time-course PXD040732. Annotated tandem mass spectra of Nt-acetylated peptides that were identified in the different experiments were deposited in Figshare (https://figshare.com/s/90b767af1e049649e38e).

## Supplemental data

This article contains supplemental data (86, 87, 88, 89, 90).

## Conflict of interest

The authors declare that they have no conflicts of interest with the contents of this article.
